# Structural elucidation of four fungal hydrophobins belonging to classes I and II: Results from Alphafold and accelerated molecular dynamics simulations

**DOI:** 10.1016/j.csbj.2025.03.015

**Published:** 2025-03-12

**Authors:** Derrick Agwora, Bonaya Gufu, Tamás Marik, Tamás Papp, Csaba Vágvölgyi, László Kredics, Chetna Tyagi

**Affiliations:** a Department of Biotechnology and Microbiology, Faculty of Science and Informatics, University of Szeged, Szeged, Hungary; b HUN-REN-SZTE Fungal Pathomechanisms Research Group, University of Szeged, Szeged, Hungary

**Keywords:** Hydrophobins, AlphaFold, Accelerated molecular dynamics simulations, Disorder

## Abstract

Hydrophobins (HFB) find application in various industries including biotechnology and medical devices; therefore, it is imperative to elucidate and learn more about their folded structures. Few fungal HFB protein structures are available in the Protein Data Bank (PDB), and fewer have been elucidated using homology modeling or short molecular dynamics (MD) simulations in the literature. Moreover, many homology modeling algorithms will only model the region with sequence identity. Therefore, we turned towards the state-of-the-art, artificial intelligence powered AlphaFold. It performed well in predicting the core β-barrel, a characteristic of HFBs, except for HFB9A which was unfolded with low confidence scores. These initial structures were then prepared for accelerated MD simulation in the hope of observing higher protein folding. With 500 ns long aMD simulations, we were able to obtain folded and energetically stable conformations for all the proteins except HFB9A, which exhibited much higher disorder, connected with higher atomic fluctuation, lowest hydrophobicity and overall compactness, and lesser secondary structure formation as visualized during the aMD simulation. The underlying intrinsic disorder in the HFBs was found to be the basis of harder-to-reach folding by AF2 which can be compensated by enhanced sampling MD simulations like the aMD technique. The characteristic two disordered loops of class I HFBs were obtained for SC3, while HFB9A showed that not all class I HFBs contain them. Class II HFBs were more stable, folded and compact with secondary structure motifs conserved throughout the trajectory which can be correlated with their comparatively much lower intrinsic disorder.

## Graphical Abstract

**Figure:**
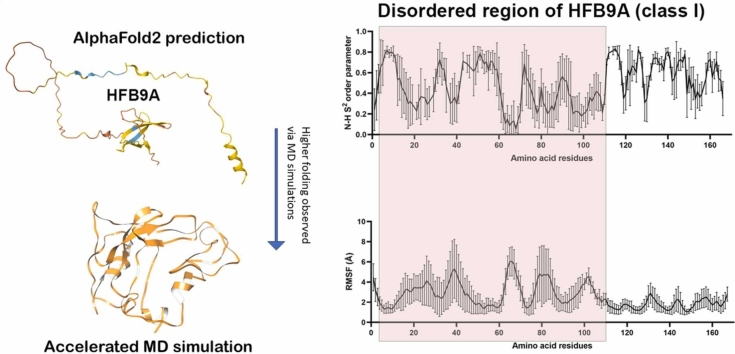

## 1 Introduction

Hydrophobins (HFBs) are a diverse group of small, cysteine-rich proteins 7–15 kDa in size, and are primarily secreted by filamentous fungi. They are notable for their unique characteristics: long disordered loops, and an exposed hydrophobic patch. HFBs play essential roles in fungal growth and development, including the formation of hydrophobic aerial structures like hyphae, spores, and fruiting bodies; aiding hyphal attachment to hydrophobic surfaces and acting as signaling molecules [42], [62], [63]. One of the most remarkable features of HFBs is their ability to spontaneously assemble into amphipathic monolayers at the interfaces of hydrophobic and hydrophilic regions [57]. The term *hydrophobin* was first introduced by Rosenberg & Kjelleberg [49] to describe bacterial cell wall components contributing to increased hydrophobicity. However, it was not until 1991 that Wessels and colleagues applied the term to describe similar proteins in fungi. Since then, the term has been predominantly used to refer to fungal proteins. Although HFBs do not exhibit significant sequence conservation, they consistently feature eight cysteine residues, including two pairs of vicinal cysteines [35].

HFBs are classified into two types based on their biophysical properties, Class I and II. Class I HFBs are typically longer, consisting of 100–125 amino acid residues, and they may undergo glycosylation. In contrast, Class II HFBs are shorter, containing 50–100 amino acid residues [24]. The critical structural feature common to both Class I and Class II HFBs is the formation of four disulfide bridges, involving cysteine pairs (1−6), (2−5), (3−4), and (7−8), with hydrophobic patch being formed between cysteine pairs (3−4) and (7−8). On the other hand, a stark difference between Class I and Class II HFBs is the presence of two disordered loops in the former, leading some to hypothesize that these loops could be the cause of ordered rodlets formed by Class I HFBs and not Class II [30]. Class I HFBs are found in both ascomycetes and basidiomycetes, while Class II HFBs are exclusive to ascomycetes [35].

Since the discovery of HFBs by Wessels et al. [62], various experimental and computational techniques have been employed to elucidate their structures and potential functions. These include: NMR, X-ray crystallography, homology modeling, and molecular dynamics (MD) simulations. One of the earlier structures determined was that of HFBII from *Trichoderma reesei*, resolved using X-ray crystallography by Hakanpää et al. [20] at 1.0 Å resolution. It was revealed that the protein has a globular structure, stabilized by a network of disulfide bonds. On the protein's surface, they identified a distinct patch of aliphatic side chains that are conserved in similar proteins. They were also the first to propose that this patch confers amphiphilic and hence surfactant characteristics. Besides this, an earlier experiment on EAS (gene *eas*) from *Neurospora crassa* pointed out the intrinsic disordered nature of some HFBs in solution, and their transition to ordered functional aggregates at hydrophobic-hydrophilic interfaces [37]. Kwan et al. [30] later used X-ray fiber diffraction to show that these aggregates formed by EAS contain a repeating β-structure. Their experiment supported earlier circular dichroism (CD) data, attenuated-total-reflectance–Fourier transform infrared (ATR-FTIR) data, and fiber diffraction data which indicated an increase in β-structure upon polymerization [30].

Computational techniques have also not been left behind. Homology modeling, for instance, has been employed by Li et al. [34] to determine the structures of HFBs from the edible mushroom *Cordyceps militaris*. The predicted structures are very characteristic of HFBs including hydrophobic patches, at least two α-helixes and a β-barrel. Besides homology modeling, even as early as 2002, MD simulations were being performed on SC3 - the benchmark hydrophobin in this work [64]. That study showed that the formation of secondary structures, primarily β-sheets, occurred significantly faster when the protein folded at the interface compared to folding in the bulk solvent. The structure for HFB7, another protein selected for this study has also been predicted using both homology modeling and MD simulations [46]. The interesting result of this study was that they found HFB7 to contain two exposed hydrophobic patches.

Our goal was to identify theoretical methods capable of accurately elucidating the structures of HFBs. To achieve this, we selected SC3, a well-characterized Class I HFB, for comparison with previous studies, alongside HFB9A, a less-studied Class I HFB. Similarly, for the representation of class II HFBs, we selected HFB2–6 which has been modelled before, and HFB7, a novel orphan HFB found only in few clades of *Trichoderma*. We first utilized AlphaFold2 (AF2) to obtain initial structures and then applied accelerated molecular dynamics (aMD) simulations to elucidate the structures and folding dynamics of HFBs. At the core of AF is a deep convolutional neural network, that as input takes the sequence of the query protein, plus statistics based on its Multiple Sequence Alignments (MSA) [52]. Using this it predicts the three-dimensional protein structure. AF leverages the insight that residues in close spatial proximity tend to exhibit correlated mutation patterns [28]. When studying the folding of eight helical proteins in water, Duan et al. [11], achieved folded structures within 40–180 ns into the simulation, not so with the classical MD (cMD) simulations that they used for comparison. They also concluded that the representative structures obtained from aMD most closely resembled the native structures of the NMR-determined, helical proteins under study [8]. Our group showed that 1 µs of aMD simulations on the Alamethicin peptide, with carefully selected boost parameters, would reproduce a 2.7 µs meta trajectory utilizing gentler boosts [58]. Pierce et al. [45], showed that even without prior knowledge of the free energy landscape of a protein, successful simulations can be run with aMD. Given these strengths, aMD was seen as an attractive option. In addition, a survey of the literature shows that this technique has not yet been utilized to study HFBs. Due to the various biotechnological applications of HFBs, it is imperative to study every aspect of their structure, function and potential applications. For example, HFB coatings on biosensors and electrodes have the potential to enhance surface hydrophobicity, thereby preventing denaturation and hence maintaining long-term activity [59]. HFBs also hold promise for enhancing the biocompatibility of medical implants, as they do not elicit immunogenic reactions [24]. Class II HFBs have been employed to stimulate cell growth on solid surfaces [25]. The self-assembly property of HFBs has been harnessed to formulate water-insoluble drugs for oral administration, resulting in increased bioavailability for certain drugs [60].

## 2 Methodology

### 2.1 Selection of hydrophobin proteins for molecular modeling studies

For our study, we selected 4 HFB proteins, 2 representing proteins from Class I and another 2 representing Class II. HFB9A from *Trichoderma harzianum* (Uniprot ID: A0A2N1L9M2, 167 a.a.) was chosen as a representative of Class I HFBs as it has not been very well-studied in terms of its folded structures and dynamics. The other Class I representative was chosen to be SC3 from *Schizophyllum commune* (Uniprot ID: P16933, 136 a.a.), as it is a well-studied protein and gives us a chance to compare our application of the new aMD technique for studying HFBs.

For Class II representation, we chose HFB2–6 from *T*. *asperellum* ACCC30536 (Uniprot ID: I7AQG9, 106 a.a.) [26] and HFB7 (Uniprot ID: A0A1P8YYD7) which is only expressed by members of the Harzianum and Virens clades of *Trichoderma* [46].

### 2.2 Initial folded protein structures used as starting points for MD simulations are obtained through AlphaFold2

For MD simulations, an initial structure in the PDB format is required. This structure can be obtained from freely available databases, such as AlphaFold (AF) DB, or modeled using online homology modeling tols like SWISS-MODEL (https://swissmodel.expasy.org/) or artificial intelligence (AI)-based tools such as AlphaFold2/3 (AF2/3 - https://alphafold.ebi.ac.uk/).

In this work, both the Class I proteins; SC3 and HFB9A were modeled using AF2 via Google’s Colabfold server [41]. While the already predicted class II proteins: HFB2–6 and HFB7 were downloaded from the Alphafold Database. AF3 is the current version of AlphaFold, therefore, we also recently re-modelled all structures just to see if any large differences are seen but there are virtually no differences from the structures modelled with the AF2 version. For decades, predicting protein structures has posed a formidable challenge despite the presence of various methodologies such as nuclear magnetic resonance (NMR), X-ray crystallography, and cryo-electron microscopy (cryo-EM). These conventional techniques have only unraveled structures for approximately 200,000 proteins, representing a minute fraction of the protein landscape.

### 2.3 Folding simulations of HFBs using aMD techniques

As classical MD offers limited utility in terms of shorter time scales, a relatively newer approach named aMD was adopted for this study to enhance sampling. The bias potential function, introduced by Hamelberg et al. [23], [22], was applied to make the simulation “jump over” high energy barriers and to sample rare events.

1. System building: The initial conformations of HFB proteins obtained from AF2 were prepared using ‘tleap’ module of AmberTools18 and solvated in water (TIP3P model) as the solvent. 14122 water residues were added for SC3 while 48584 residues were added for HFB9A due to the large coverage of the coordinate space. Similarly, 13310 water residues were added in the HFB2–6 system while only 7585 residues were added in the HFB7 system. The Amberff19SB force field was utilized to prepare all the systems.
2. Iteration of minimization and equilibration steps while slowly lowering restraints: All systems were prepared for aMD in six consecutive steps including minimization, heating and relaxing i.e. (a) minimization of only water residues (steepest descent method for the first 100,000 cycles and then shifting to the conjugate gradient algorithm) of solvent for 300,000 cycles while keeping the protein under restraint, (b) relax water by movement at 300 K under isothermal and isobaric (NPT) conditions while keeping the protein under restraint, (c) minimization of the whole system for 500,000 cycles, (d) heating from 0 K to 300 K under isothermal and isovolumetric (NVT) conditions while keeping the protein under restraints, (e) relax the system at 300 K for 0.6 ns while keeping the heavy atoms of the protein under restraint, and (f) relax system at 300 K under NPT conditions for 1 ns with no restraints. SHAKE bond length constraints were applied on all bonds involving hydrogen. The temperature scaling was carried out using Langevin thermostat while the pressure was regulated using the default Berendsen barostat for all corresponding calculations.
3. Classical MD (Production run): The system equilibration steps are followed by a short classical MD run to obtain average dihedral and potential energies (kcal mol^−1^) for 2 ns at 300 K temperature and periodic boundary condition was used with constant pressure using Berendsen barostat. Once this classical MD production run is completed, we can calculate boost parameters.
  The boost parameters E_dihed_, α_dihed_, E_total_ and α_total_ were calculated as required in the equation below:
  E_dihed_ = V_avg_dihed_ + a1 × N_res_, α_dihed_ = a2 × N_res_/5;
  E_total_ = V_avg_total_ + b1 × N_atoms_, α_total_ = b2 × N_atoms_
  where N_res_ is the number of amino acid residues, N_atoms_ is the total number of atoms in the system. V_avg_dihed_ and V_avg_total_ are average dihedral and total potential energies.
4. aMD simulation: The classical MD production run is followed by aMD simulations which were carried out for 500 ns for each protein at 300 K temperature, 2 fs time step, and energies and boost information were written at every 1000 steps in the ‘amd.log’ file marked as default. The electrostatic interactions were calculated using particle mesh Ewald method (PME) [9] and long-range interactions were also calculated with cutoff of 10.0 Å. The temperature scaling was carried out using Langevin thermostat without pressure scaling under periodic boundary conditions during aMD. The GPU machines available through the Komondor supercomputer (https://hpc.kifu.hu/hu/komondor) were utilized for all aMD simulations. All simulations were carried out using “pmemd.cuda” implementation of Amber22 [4], also available at the cluster. aMD can be carried out using three criteria, i) independently boosting the torsional terms of the potential (iamd = 2) or ii) the whole potential at once (iamd = 1), and iii) to boost the whole potential with an extra boost to torsions (iamd = 3). The third criterion was chosen for the reason that it gives an option of better reweighting distribution [61], in as much as dihedral-only aMD enhances the convergence of the underlying FEL 5-fold vis-à-vis cMD.
5. Analysis (Reweighting): An important aspect of an aMD calculation is to reweight the distribution to remove the effect of boost applied to the system and to recover the original free energy landscapes. To recover this distribution, ‘amd.log’ is generated during the run that contains information about the extra boost added to each snapshot of the trajectory. The usual “exponential average” method suffers from high statistical noise which was overcome using a method named Maclaurin series expansion, which approximates the exponential Boltzmann factor and reduces energetic noise considerably [40]. Therefore, Maclaurin series expansion up to the 10th order was found to be the most accurate reweighting procedure for all simulations in this study.
6. Analysis (clustering): As per [2], have detailed the method of their clustering procedure incorporated with ‘grcarma’, the tool that we used for carrying out dihedral principal component analysis (dPCA) based clustering. To identify isolated peaks in the density distributions calculated from dPCA, the algorithm calculates a threshold value for density above which the peaks can be defined. They have implemented an automatic procedure by applying a ‘variance-explained criterion’. It tests a large number of threshold values starting from very high values and gradually decreasing until the mean density of the map has been reached. For each calculated threshold, the percentage of the map’s variance is calculated explained by the peaks above the threshold. They aim for a threshold value that can explain at least 80 % of the original PC map’s variance. The authors also outlined the possible limitations; “Depending on the peak-picking threshold selected by the variance-explained criterion, low lying peaks may escape detection or closely related -but otherwise distinct- conformations may be assigned to the same cluster”. Therefore, the FEL plots are automatically generated based on dPCA and the corresponding clusters along with their representative structures are also generated using ‘grcarma’. Using a python script available via [40], the PCs are reweighted and the FEL plots are recreated.
7. Analysis (Disorder): For root-mean-square fluctuation (RMSF), Lipari Szabo order parameters (S^2^), the error bars were calculated by splicing the 500 ns trajectory into 5 blocks (100 ns block each) and plotting the standard deviation from the mean. The calculation of Lipari Szabo order parameters (S^2^) was carried out using the ‘ired’ (isotropic reorientational eigenmode dynamics analysis using given IRED vectors) tool from cpptraj [48] where we provide the N-H bond vectors for every amino acid residue in the given protein. The proline amino acid residues were skipped due to non-availability of free N-H atoms at the peptide bond.

## 3 Results and discussion

### 3.1 AlphaFold2-based structure prediction of HFB9A and SC3

**Fig. 1:**
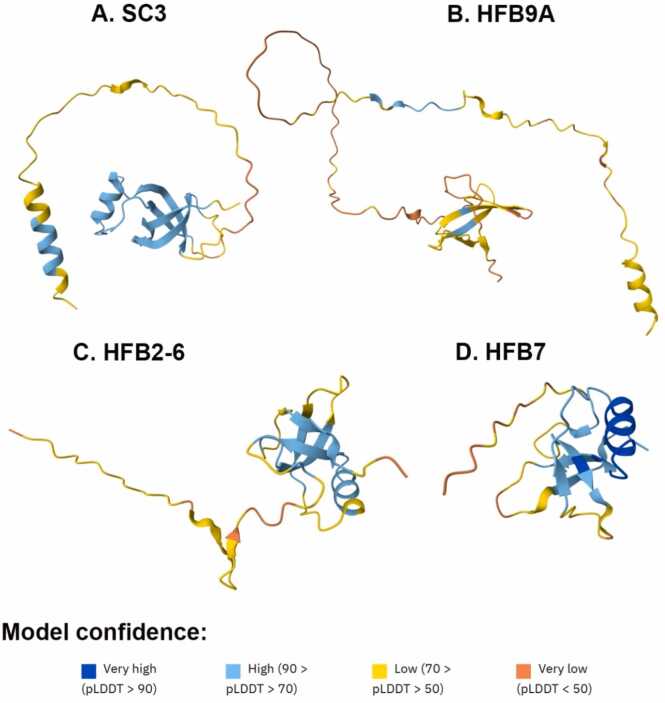
The top scoring structures obtained by AF2 for class I HFBs, SC3 (a) and HFB9A (b), and class II HFBs, HFB2–6 (c) and HFB7 (d). All of them have been colored according to their confidence scores (plDDT score).

HFB9A and SC3 were chosen for modeling since their structures could not be found from the AF2 database. As evident from Fig. 1(a and b), the characteristic β-barrel structure of SC3 was accurately predicted, with a high confidence score or plDDT score, as are its two α-helices. The structure so derived is quintessentially a hydrophobin. However, the loop spanning Leu19 up to Thr58 is quite unstructured with low plDDT scores between 50 and 70. On the other hand, the overall structure of HFB9A was quite unstructured and unfolded with very low plDDT scores. The structures of Class II proteins HFB2–6 and HFB7 were already available in the AlphaFold database and the PDB files were available as shown in Fig. 1c and d. The core of these two proteins seems to be quite similar to expected HFB characteristics but the N-terminal regions of both are unfolded with low plDDT scores. All four proteins were prepared for modelling using aMD simulations.

### 3.2 Stability of protein folding based on root-mean-square fluctuation (RMSF) as obtained from aMD simulations

All initial structures obtained from AF2 were then prepared for aMD simulations of 500 ns time-length each. The various results from aMD simulations are discussed from here on. The Root Mean Square Fluctuation (RMSF) quantifies the average displacement of a specific atom or group of atoms in comparison to a reference structure, averaged across all the atoms involved. As such, it can be used to indicate which residues have the most flexibility or mobility. These regions may indicate towards regions of domain movement which might be functionally important. We calculated RMSF for each 100 ns long block of the total trajectory and plotted the mean with error bars indicating the large structural change observed during the simulations.

**Fig. 2:**
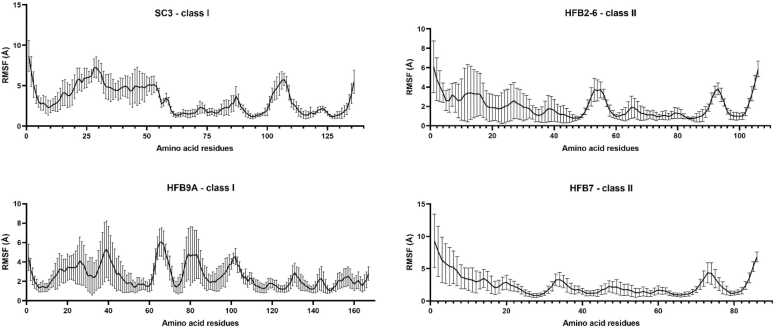
Root-mean-square fluctuation or average atomic fluctuation of amino acid residues of SC3 and HFB9A from class I and HFB2–6 and HFB-7 from class II during the 500 ns long simulation. The class II proteins show comparatively less fluctuation on the atomic level and maybe correlated with being less disordered in comparison to class I HFBs. The error bars indicates that the N-terminal half is quite more fluctuating than the C-terminal half of all 4 HFBs during the course of simulation.

HFBs have been reported to be usually unstructured as monomers in solution, for example, EAS from *N*. *crassa* adopts a more ordered β-sheet structure only upon polymerization [36]. The disorder-to-order transition drives polymerization as shown in case of EAS. Therefore, the study of their stability or conversely, their flexibility is crucial to understanding overall behavior. For both Class 1 HFBs, the fluctuation of the N-terminal region is higher than that of the rest of the protein along with higher standard deviation ranges. The two highly fluctuating loops of SC3 from residues 20–50 and 100–120 are reported for other well-studied class I proteins as the ‘disordered loops’. However, HFB9A, despite belonging to class I does not show the same pattern (Fig. 2).

**Fig. 3:**
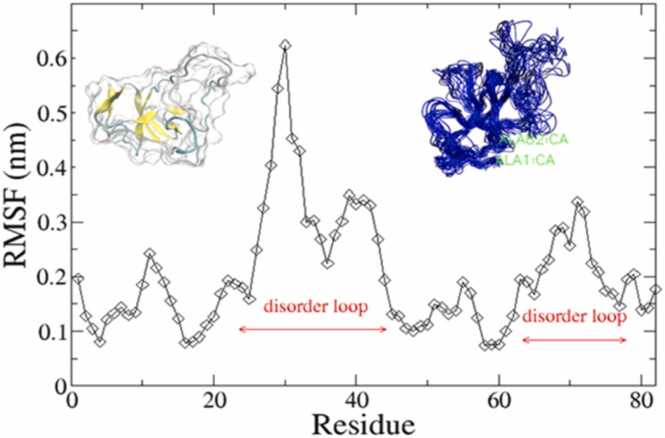
RMSF plot for a 10 ns long MD simulation of EAS.

In Fig. 3, the left subfigure is the NMR structure of EAS while the right one is the superimposition of 20 structures taken at different time intervals during the simulation (illustration adopted from the doctoral dissertation of Chen Yuwu, [7] wherein the right subfigure reproduced with permission from Elsevier [29]). The RMSF plot for SC3 and EAS share two maxima, and are quite similar, this can be attributed to the fact that they are both Class Ia HFBs. However, the RMSF plot for HFB9A does not show the presence of C-terminal disorder loop. Taylor et al. [56] also reported a folded structure based on homology modeling using I-TASSER along with another similar class I HFB, TASHYD1 from *T. asperellum* (Uniprot: Q15K91), where the β-sheet core was correctly predicted for both. Currently, the Uniprot entry for TASHYD1 shows a predicted structure with low confidence scores for the whole sequence calculated using AlphaFold. The disorder prediction of the TASHYD1 sequence also does not show the presence of 2 disordered loops as seen for SC3 or EAS. It can be concluded that not all class I HFBs contain two distinct disordered loops. Furthermore, wettability experiments performed by Bonazza et al. [3] on the hydrophobic surface of highly ordered pyrolytic graphite (HOPG) found that HFB9A did significantly increase its wettability, also in line with another property of HFBs. We could compare the very high structural degree of disorder of HFB9A with other intrinsically disordered stress response proteins including dehydrins, DprA of *Aspergillus fumigatus* which are hydrophilic [17]. Interestingly, dehydrins also exhibit disorder-to-order structural transition, upon binding to membranes, just as has been demonstrated for HFBs. This transition takes place with an increase in α-helicity, as has also been observed for SC3.

On the other hand, Class II protein HFB2–6, shows higher fluctuation at the N-terminus with considerable deviation from the mean. But overall, the mean values remain under 4 Å for most of the sequence length. HFB7 has the lowest RMSF in contrast to all the other HFBs, apart from its flanking N and C termini. Most of the sequence length shows fluctuation values under 5 Å with minor deviations. Overall, it seems that Class II HFBs have less atomic fluctuation of the backbone with low ranges of standard deviation from the mean, therefore, are better able to maintain their conformation (Fig. 2).

Indeed, Class I Cys3-Cys4 loops are more extended than class II loops [35], this explains the large N-termini fluctuations of class I HFBs. The flexibility and fluctuations of these loops are of experimental consequence. Niu et al. [43] replaced the Cys3–Cys4 loop of Class I HGFI with that of Class II HFBI. The replacement of this loop led to the formation of less stable amphipathic membranes at hydrophilic-hydrophobic interfaces. In addition, the self-assembly was not accompanied by an increase in β-sheet formation, and neither were typical rodlet structures observed. A similar observation was reported by Schulz et al. [51] when they studied the mineralization properties of HFBs and chose EAS as Class I representative and HFBII as representative of Class II. EAS, which can induce mineralization during emulsion stabilization, underwent a large conformational change during the MD simulation at the hexane-water interface in the presence as well as absence of ions. On the other hand, HFBII which does not induce mineralization, only slightly changed its structure during adsorption at water/hexane interface and upon addition of ions. We can infer that highly fluctuating regions in HFBs confer an ability to transform their shape easily, which is directly related to many of their functions just like in the previous example. As such, one may conclude that the highly extended and highly fluctuating loops of Class I HFBs play a pivotal role in rodlet structure formation. Therefore, RMSF profiles obtained via MD simulations could be useful in the characterization of HFBs before studying them during wet-lab experimentation.

### 3.3 Evolution of secondary structure of all 4 proteins as a function of simulation time

**Fig. 4:**
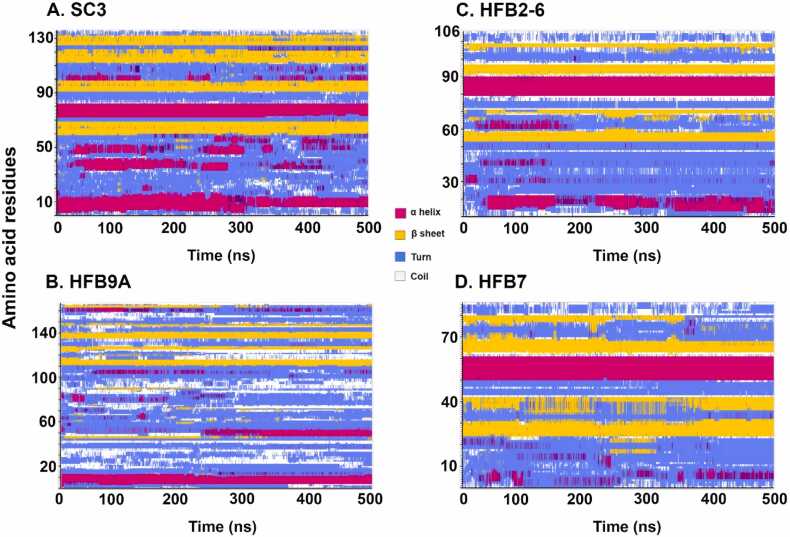
Secondary structure evolution of (a) SC3 protein (136 aa residues), (b) HFB9A protein (167 aa residues), (c) HFB2–6 protein (106 aa residues), and (d) HFB7 protein (86 aa residues) as a function of simulation time.

**Fig. 5:**
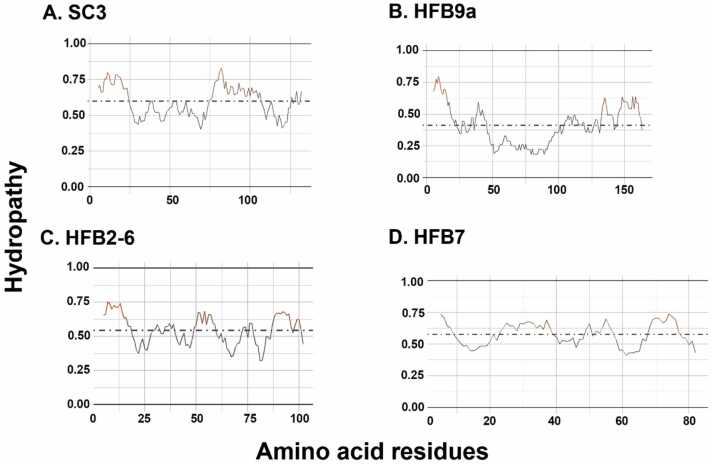
Hydropathy profiles of Class I HFB proteins SC3 (A) and HFB9A (B), and Class II HFB proteins HFB2–6 (C) and HFB7 (D). The Kyte and Doolittle measurement of hydropathy [31] is used, scaled with Arg having a hydropathy of 0.00 and Ile having a hydropathy of 1.0. In this scale, hydrophilic amino acids would have values closer to 0.00 while hydrophobic amino acids would have values closer to 1.

Fig. 5a shows the time evolution of the secondary structure of the SC3 protein where the different colors signify secondary structural elements. From the figure it is apparent that the β-sheet character from residues 60–70 and last three sheets which forms part of the β-barrel are quite stable throughout the simulation (Fig. 4a). The α-helix character at the N-terminus and from residues 73–82 also remains conserved throughout the simulation. These two helices are always parallel to each other in the folded SC3 conformation. On the other hand, the region from residues 30–50 fluctuates between helix and/or turn character, which shows that the protein may exist in multiple states depending on the environment. Interestingly, the same region corresponds to the region of highest atomic fluctuation as per the RMSF plot as seen in Fig. 2. Interestingly, Zangi et al. [64] had reported short (100 ns) classical MD simulations of SC3 in bulk water and hexane and also on the interface of water/hexane. The secondary structure evolution suggested that SC3 remained mostly disordered in each bulk solvent but acquired an ordered, largely β-sheet character when placed at the water/hexane interface.

As apparent from Fig. 4b, a complete β-barrel could not be observed throughout the simulation, with only small regions from residues 110–140 showing β-sheet formation for HFB9A. The N-terminal α-helix is conserved, just as observed for SC3, but HFB9A lacks the formation of the second helix until 300 ns. The elongation of the simulation time to 500 ns revealed the formation of the second helix between residues 40–50 which also correlates with low-fluctuation region observed from RMSF plot for HFB9A (in Fig. 2). This protein has much more turn/coil formation than observed for other class I HFBs. Surprisingly, HFB9A – although classified as Class I HFB – does not show usual structural characteristics of Class I proteins.

From Figs. 4c and 4d it is evident that the time-evolutions of secondary structure for Class II HFBs are remarkably similar to each other, as opposed to the differences that can be seen between the two selected Class I HFBs. A possible explanation for this would be the presence of shorter disordered loops in Class II HFBs, in contrast with the longer loops of Class I HFBs. Longer loops, in principle, would be more susceptible to greater structural flexibility [35]. Another possible reason could be the fact that Class II HFBs are exclusively found in Ascomyceta, forming a uniform phylogenetic group, and their sequences show higher similarity, ranging from 29 to 95 percent [35]. On the other hand, Class I HFBs are found in both Ascomycota and Basidiomycota, and have more sequence variation with a wider distribution of sequence lengths [35].

We also calculated the average hydropathicity for the whole sequence as shown in Fig. 5 to draw correlations with the observed fluctuating (disordered) regions and secondary structure evolution. As evident from the figure, the average hydropathicity values of the two Class I proteins differ quite a lot where for SC3, highly hydrophobic character can be seen. The average scaled hydropathy for SC3 is 0.586, showing that it is more hydrophobic than HFB9A with an averaged scaled hydropathy of 0.412 (Figs. 5a and 5b). Zhao et al. [65] suggested lower hydrophobicity of HFB9A/HFB9B proteins based on their primary structure analysis. It can also be noted that for SC3, the more hydrophilic regions match the most fluctuating regions (2 disordered loops) as per the RMSF plots in Fig. 2.

HFB9A has been found not to play a role in the adhesion of hyphae to roots but rather to aid in colonizing the internal parts of roots post-adhesion [56]. It would be interesting to investigate in the future whether this divergence in behavior from other Class I proteins, which are primarily implicated in adhesion, is due to its relatively higher hydrophilicity as compared to the other Class I HFBs. The higher hydrophilicity of HFB9A-coated hyphae could interact favorably with the internal parts of the plant root. Zhao et al. [65] calculated the hydropathy scores of 11 HBFs when comparing between families of surface-active proteins. Of these, HFB9a resulted in a GRAVY score of −0.8, the highest hydrophilicity value amongst the sampled HFBs. However, Taylor et al. [56] found that HFB9A mutants had significantly lower hyphal surface hydrophobicity relative to the wild type. Their results indicate that despite the relative lower hydrophobicity of HFB9A in comparison to other HFBs, they still contribute to surface hydrophobicity – a general property of HFBs.

**Fig. 6:**
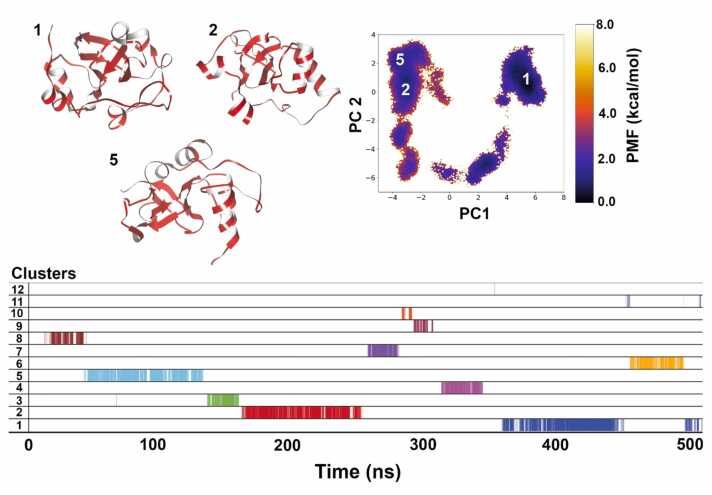
Dihedral PCA-based reweighted free energy landscape of SC3 simulation shows 4 representative structures belonging to separate minimum energy basins marked as 1, 2, and 5. Overall, 12 clusters were obtained from the 500 ns long aMD simulation and clusters 1, 2 and 5 occupy the majority of simulation time.

While on the whole, both proteins from class II are slightly hydrophobic, there are clear differences in position-specific hydrophobicity values. The first ten residues of HFB2–6 are more hydrophobic than those of HFB7 (Figs. 6c and 6d). In addition, these ten residues in HFB2–6 (Fig. 4c) exhibit more interconversion to α-helical structure than in HFB7 (Fig. 4d). The average scaled hydropathy for HFB2–6 is 0.542, showing that it is slightly less hydrophobic than HFB7 with an averaged scaled hydropathy of 0.576. Overall, the results indicate that regions of higher hydrophobicity may drive stricter secondary structural folding and consecutively show lesser fluctuation or disorder in case of HFBs.

### 3.4 Dihedral PCA-based reweighted free energy landscapes

The analysis of simulation results was carried out using Principal Component Analysis (PCA) to condense the dimensionality of data and offer a visual representation of the free-energy landscapes. This approach allowed for the identification of intermediate states and elucidated the pathways leading to the final folded state [38]. However, employing Cartesian coordinate PCA (cPCA), which considers overall peptide motions, could potentially obscure the clarity of free-energy basin distributions [55]. To address this, dihedral-angle-based PCA (dPCA) [1] was utilized, focusing solely on internal motions characterized by ϕ and ψ dihedral angles during the peptide folding process. Analyzing the free energy landscape (FEL) through internal motions, projected along the first two principal components (PC1 and PC2) using the equation μ (q1, q2) = −kBT lnP (q1, q2), yields precise insights into the locations of minimum energy wells and the barriers separating them. In the free energy landscape, the darkest violet regions denote the lowest energy minimum.

For SC3, a total of 12 clusters were obtained mapped as shown in Fig. 6. The first cluster is the darkest colored region on the plot i.e. ∼ 0 kcal/mol energy minima level and occurs at the end of the trajectory. It makes up 10 % of the whole simulation time while the second cluster was found to be the most populated by a small margin (11 %) and the fifth cluster makes up 10 % and is the third most populated cluster. All 3 conformations of SC3 obtained during dPCA-based clustering are not very different from each other in terms of the protein core including the β-barrel and presence of α-helices but differ mostly in the loop regions. This core structure is stabilized early in the simulation as evident from the placement of cluster 5 on the cluster plot (Fig. 6). Cluster 2 energy basin lies close to it in the FEL plot and also occurs after cluster 5. In an attempt to show degree of structural convergence, we employed what are described as self-consistency checks (SCC) adopted from Sawle and Ghosh [50]. The number of clusters obtained as the simulation progresses i.e. dPCA based clustering was calculated for every 100 ns increment of the total 500 ns trajectory. The number of clusters obtained for each incremental block is provided in Supplementary information (Figure S1). The simulation is said to satisfy this SCC if the graph plateaus which could be observed for SC3. From 17 clusters obtained at 300 ns, it plateaus at 12 clusters obtained at 400 and 500 ns (refer to Figure S1). In other words, no new structurally distinct cluster is obtained even when the enhanced sampling simulation progresses indicating convergence.

**Fig. 7:**
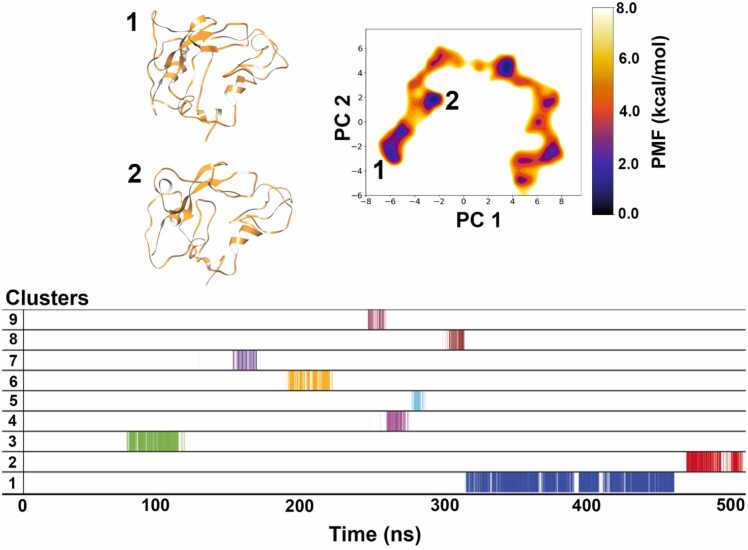
Dihedral PCA-based reweighted free energy landscape of HFB9A simulation shows first two representative structures belonging to separate minimum energy basins marked as 1, and 2. Overall, 9 clusters were obtained from the 500 ns long aMD simulation and the first 2 clusters occupy the majority of simulation time.

For HFB9A as shown in Fig. 7, nine clusters were obtained, and of these the first cluster is the most populated, making up 16.5 % of the whole simulation time. The second cluster makes up 3.2 % of the whole simulation. The top 2 clusters lie slightly separated from each other as shown in the FEL plot but appear after each other as can be seen in the cluster plot. If we compare with the representative structures, we will find that none of the conformations show a strict protein core folding as observed for SC3. Cluster 1 representation shows the formation of some secondary structure with a certain degree of compactness when compared to the structure obtained from AF2. This structure seems to be highly disordered; one cause could be simulation of a hydrophobic protein in water solvent, but we could observe that HFB9A is less hydrophobic than SC3, therefore, this degree of disorder must be intrinsic and not an effect of simulations in water solvent. Despite indications of intrinsic disorder, the structural convergence SCC shows the number of clusters plateau after 300 ns which means that no new distinct states were discovered for the following 200 ns long biased simulation (Figure S1).

**Fig. 8:**
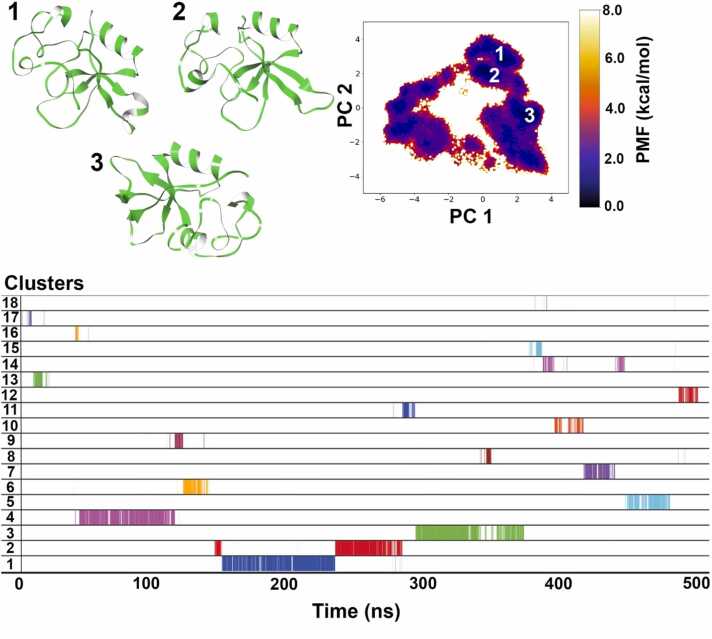
Dihedral PCA-based reweighted free energy landscape of HFB2–6 simulation shows 3 representative structures belonging to separate minimum energy basins marked as 1, 2 and 3. Overall, 6 clusters were obtained from the 500 ns long aMD simulation and the first 3 clusters occupy the majority of simulation time.

For HFB2–6 as shown in Fig. 8, a total of 18 clusters were obtained. The first cluster is the most populated and makes up 10 % of the total simulation time, while the second and third clusters make up 7.2 % and 8.4 % respectively. Clusters 1, 2 and 3 form the 3 lowest energy basins (darkest blue color basins) on the FEL plot and their representative structures show high degree of folding and compactness. Huang et al. [26] made use of the SWISS-MODEL program that predicted that the three-dimensional structure of HFB2–6 would be a compact globular shape. Their predicted structure comprises of two neighboring β-hairpins forming the core of the β-barrel. Additionally, there is an α-helix connected to the surface of the β-barrel via a disulfide bond, and another disulfide bond links two strands within each β-hairpin. Disulfide bonds are positioned at both ends of the barrel structure, ensuring a high level of stability for HFB2–6. The AF2-predicted structure does not have a full β-barrel, and only two β-sheets can be seen. It is also interesting to note, that both top representative structures have a half β-barrel formation. This partially formed β-barrel is similar to the *N. crassa* HFB NC2 resolved using solution NMR [47], which suggests that not all HFBs form fully developed β-barrel structures. The NC2 structure, as revealed by solution NMR spectroscopy, consists of four β-strands, arranged in an antiparallel orientation. Together, they form an open or semi-barrel shape. Moreover, a single helix is located on the surface of this barrel-like structure. The regions between the β-sheets contain two loops that lack a consistent secondary structure.

Surprisingly, HFB2–6 failed the SCC criteria for convergence even though it indicates comparatively less disorder, higher secondary structural folding and overall compactness during the simulation. The number of clusters obtained increased dramatically from 10 clusters obtained at 400 ns to 18 clusters obtained when the whole 500 ns trajectory is considered (Figure S1). As true convergence from MD simulations is a theoretical concept and only one criterion cannot always answer convergence of structure, we employed another method based on probability theory known as Good-Turing formalism and reported by Koukos & Glykos [27]. It calculates the root-mean-square-deviation (RMSD) matrix and can predict that how distinct of a structure could be obtained if the simulation time were doubled. In other words, this analysis indicates the probability of observing a molecular configuration not observed before [53]. Based on the results, it can be said that the expected maximal RMSD value upon doubling the simulation time will not be more than 6.4 Å. Another way to understand the results is for example, one out of every five (probability of unobserved species, P_unobs = 0.20) new structures encountered will differ by an RMSD of at least 2.3 Å upon increase in simulation time (Figure S2). Because the input data for this prediction also includes unfolded conformations in the beginning of the simulation, it is not illogical to connect the high predicted RMSD value (6.4 Å) to their presence in the sampling cohort. The same analysis was carried out for class I HFBs, SC3 and HFB9A where the results showed non-convergence of trajectory based on RMSD matrix-based prediction of unobserved species.

**Fig. 9:**
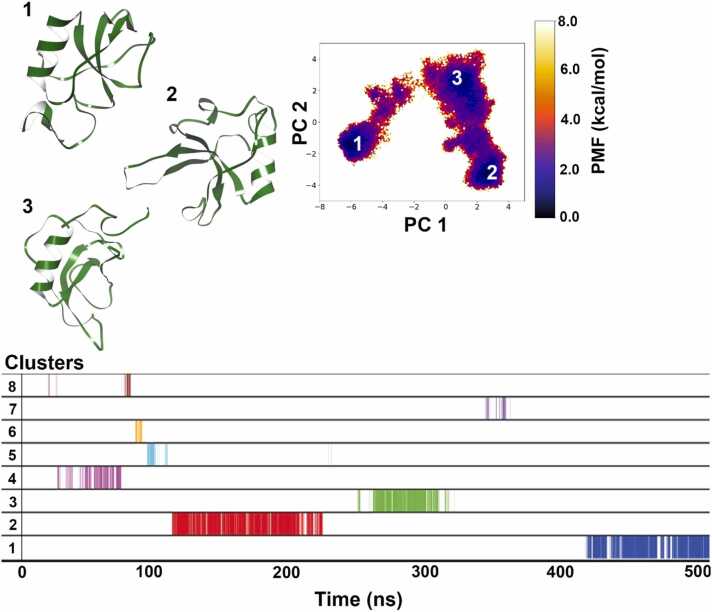
Dihedral PCA-based reweighted free energy landscape of HFB-7 simulation shows 2 representative structures belonging to separate minimum energy basins marked as 1 and 2. Overall, 8 clusters were obtained from the 500 ns long aMD simulation and the first 3 clusters occupy the majority of simulation time.

For HFB7 as shown in Fig. 9, a total of 8 clusters were obtained with the 2nd cluster most populated, and makes up 14 % of the whole simulation time followed by cluster 1 with 11.4 % occupancy and cluster 3 with only 5.7 % occupancy. The representative structures of clusters 1 and 2 shows formation of a half ß-barrel and a strict α-helix in the vicinity just like in case of HFB2–6. These two clusters form distinct energy basins (∼ 0 kcal/mol) on the FEL plot where cluster 2 occurs early in the simulation (from 110 – 210 ns) and cluster 1 occurs at the end of simulation (after 400 ns till the end of trajectory). This indicates that the core secondary structure of HFB7 is obtained early during the folding process and remains intact. This was also observed during the SCC convergence criteria where the number of clusters line plateaus after 300 ns and does not show much variance overall (Figure S1). It also showed better convergence results than observed for HFB2–6 based on the Good-Turing calculation. The expected maximal RMSD upon doubling the simulation time is 5.65 Å (Figure S2).

Przylucka et al. [46] reported the three-dimensional structure of HFB7 constructed using homology modelling with the HFB2 structure of *T*. *reesei* selected as the template (PDB ID: 2B97) with 62.3 % sequence similarity. The model with the best score was also simulated using MD techniques for 4 ns. The structure shows a well-formed β-barrel characteristic of HFB proteins. HFB7 forms a separate, taxonomically restricted occurrence in the Harzianum and Virens clades of *Trichoderma*. By using SignalP (https://services.healthtech.dtu.dk/services/SignalP-5.0/), they found that the N-terminal forms a signaling peptide of 15 amino acid residues. It forms a separate clade from other *Trichoderma* Class II HFBs. They also reported that the expression of the *hfb7* gene increased during interactions with other fungi-*Fusarium oxysporum* and *Athelia rolfsii* - and a plant - *Solanum lycopersicum* - and also in response to abiotic stress conditions. When studying the effect of HFB7 on poly(ethylene terephthalate) (PET) hydrolysis, Espino-Rammer et al. [13] found that while HFB7 reversed the wettability of PET, making it more hydrophilic and reducing the water contact angle, the water contact angles on glass, which is a hydrophilic surface, were not affected. They argued that this result may be because HFB7 was not properly folded. Therefore, studying folding dynamics of proteins with varying degrees of intrinsic disorder can aid in understanding experimental results.

### 3.5 Intrinsic disorder, folding and stability of HFBs

Hakanpää et al. [21] determined the crystal structure of the HFB1 protein by means of X-ray crystallography. They found structural flexibility in the loop regions of the native structures. When determining hydrophobins which are implicated in rodlet formation in *A. nidulans*, Grünbacher et al. [18] used the DisEMBL software (http://dis.embl.de/) to predict intrinsic ‘disorderedness’ of the structures under study. They found that in RodA, DewB and DewA HFBs, intrinsic disorder was predicted for two large loops that were situated towards the C-terminus. DewD was found to have the highest amount of disorder. Gandier et al. [15] characterized SC16 using NMR spectroscopy, and found that the second loop joining β_2_ to β_3_ was largely disordered. De Simone et al. [10], by means of extensive MD simulations of the disordered loops of EAS of *N. crassa*, concluded that the large disordered loops may have role in preventing aggregation of HFBs in solution. The above examples show that ‘disorderedness’ is a general feature of HFBs, especially in their loop regions.

Intrinsically disordered proteins (IDPs) account for 50 % of signaling-associated proteins in eukaryotes. With low sequence complexity, lack of large hydrophobic residues but full of charged and polar amino acid residues, these proteins do not have stable tertiary structures under physiological conditions. Many tests (for example: report by Eva Smorodina (https://310.ai/blog/alphafold2-alphafold-multimer-alphafold3)) have reported that even though Alphafold2 and 3 perform very well with the prediction of structures of ordered proteins, they mostly fail when it comes to IDPs, proteins with disordered regions and dynamic proteins.

Successful simulations of disordered protein ensembles have been challenging as it requires both accurate description of the conformational dependence of energy and sufficient sampling of the conformational space of the protein. Early simulations suffered from a lack of appropriate force fields and limitations in conformational sampling [6]. Lee & Chen [33] promoted the use of coarse-grained models for IDPs to improve local conformational transitions and achieve convergence on various ensemble conformational properties, however, this approach suffers from the limitation of compromise on the recovery of proper statistical ensembles at the atomic level. The sampling bottleneck is solved with the use of enhanced sampling and GPU computing both of which were employed in this work. Gaalswyk and coworkers [14] reported the need for employing self-consistency checks in all-atom MD simulations of IDPs to ensure convergence of observables. Therefore, we have reported the convergence of ‘number of clusters obtained with simulation time’ as the SCC criteria (Figure S1). Another issue that must be mentioned is that not one convergence criterion works well with all protein examples showing disorder and more than one criterion must be employed which we addressed with the Good-Turing method described previously. Class I HFBs did not satisfy the Good-Turing criteria, while class II, HFB2–6 failed the SCC criteria. Gaalswyk and coworkers [14], also reported an initial 3 µs long classical MD simulation to obtained initial representative conformations and then to run greater than 20 µs long simulations for each to satisfy multiple convergence criteria. Unfortunately, the computational infrastructure available for many academics around the world does not come close to be able to run such long simulations. Lastly, the consideration of the force fields used for MD simulations of IDPs can prove to be a limitation where many studies have been conducted to find the best bet out of the many. A recommendation from Shabane and coworkers [54] reported using Amber forcefield ff99SB in combination with OPC water model to be the most accurate for simulations of IDPs. Then Chan-Yao-Chong and coworkers [5] compared 13 force fields including Amber and CHARMM and reported that Amber ff19SB resulted in more compact conformations and higher ß-strand occupancy than ff99SB. It was clustered within the high-performing force field group based on many parameters along with charmm36, ff99SB force fields. However, another study by Pedersen and coworkers reported that ff19SB/OPC produced overly compact conformational ensembles and show discrepancies in the secondary structure compared to the experimental data [44]. We employed the latest Amber ff19SB with TIP3P model for the study of these proteins which can aid to the understanding of effect of different force fields in combination with various water models.

Firstly, we report the predicted disorder values using IUPred3. IUPred3 is a widely used tool for predicting protein disorder, built on a foundational understanding of the biophysical properties of IDPs [12], [39].

**Fig. 10:**
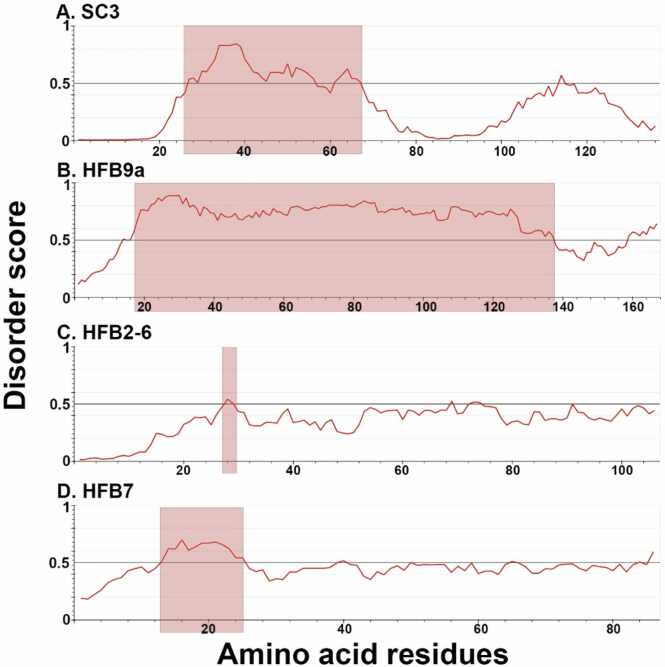
‘*IUPred3*’ plots for SC3 (A), HFB9A (B), HFB2–6 (C) and HFB7 (D). Residues with a score 0.0–0.5 are predicted to be ordered, regions 0.5–1.0 are predicted to be disordered. The algorithm was set to make global predictions of disorder, focusing particularly on identifying disordered regions spanning over 30 amino acids in length.

From the above two plots for Class I HFBs (Fig. 10a and b) it can be seen that HFB9A is predicted as disordered throughout the majority of its length except for the terminal regions. The prediction for HFB9A matches its FEL plot as per Fig. 7, where no true energy minimum is found because of its highly fluctuating regions. The N-terminal fluctuating region of SC3 can be seen to be correctly predicted as being disordered, so is the C-terminal region, though to a lesser extent overlapping with the regions of the two disordered loops. HFB9A does not show the presence of classic two disordered loops as seen for other class I HFBs. ‘IUPred3’ predicted the selected Class II HFB proteins to be more ordered than the selected Class I HFBs (Fig. 10c and d). This is in keeping with the fact that they lack the long-disordered loop regions that are present in Class I proteins. From the plots it can be seen that HFB7 is predicted to have an N-terminal ‘disordered’ region. HFB2–6 is the least ‘disordered, as per this algorithm.

**Fig. 11:**
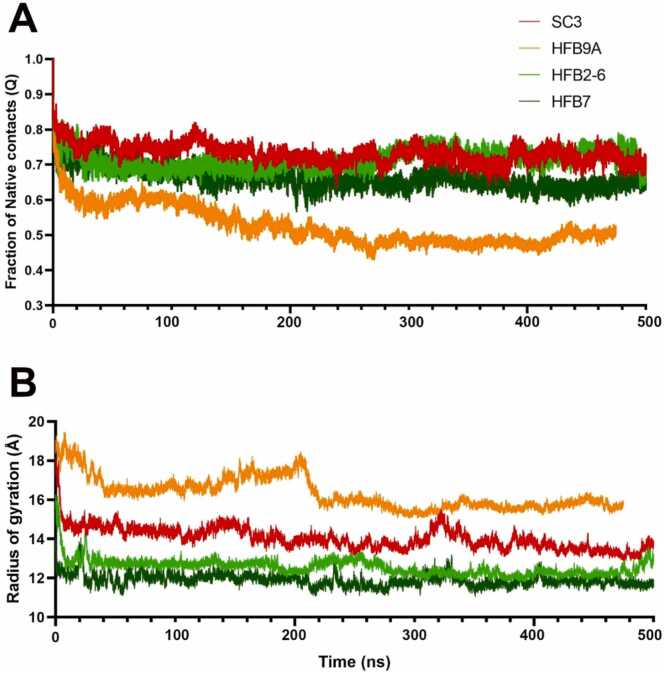
(A) Fraction of native contacts with the first frame as reference for all 4 HFBs as a function of simulation time. HFB9A shows the steepest decrease amongst others which indicates large structural shift, (B) Radius of gyration (Å) as a function of simulation time. HFB9A shows the highest RoG value which shows least degree of compactness of structure.

Furthermore, we studied disorder and overall compactness of the proteins based on two traditionally used analyses: fraction of native contacts (Qs) calculated with respect to the first frame of the simulation (Fig. 11a) and the radius of gyration (RoG in Armstrong) (Fig. 11b). HFB9A stands out in both, where fraction of contacts reduces rapidly along the simulation and has the highest RoG amongst other HFBs i.e. lesser degree of compactness and folding. As earlier discussed, Amber ff19SB/OPC can sample overly compact conformations but in this case (ff19Sb/TIP3P), the non-compact sampling of HFB9A seems like an improvement. This force field scores higher on prediction of native contacts [5]. Apart from HFB9A, all 3 other HFBs show quite a stability in maintaining native contacts throughout the simulation. RoG graph clearly shows that class II HFBs achieve more folding compactness followed by class I SC3 and HFB9A whose compactness improves considerably after 200 ns.

**Fig. 12:**
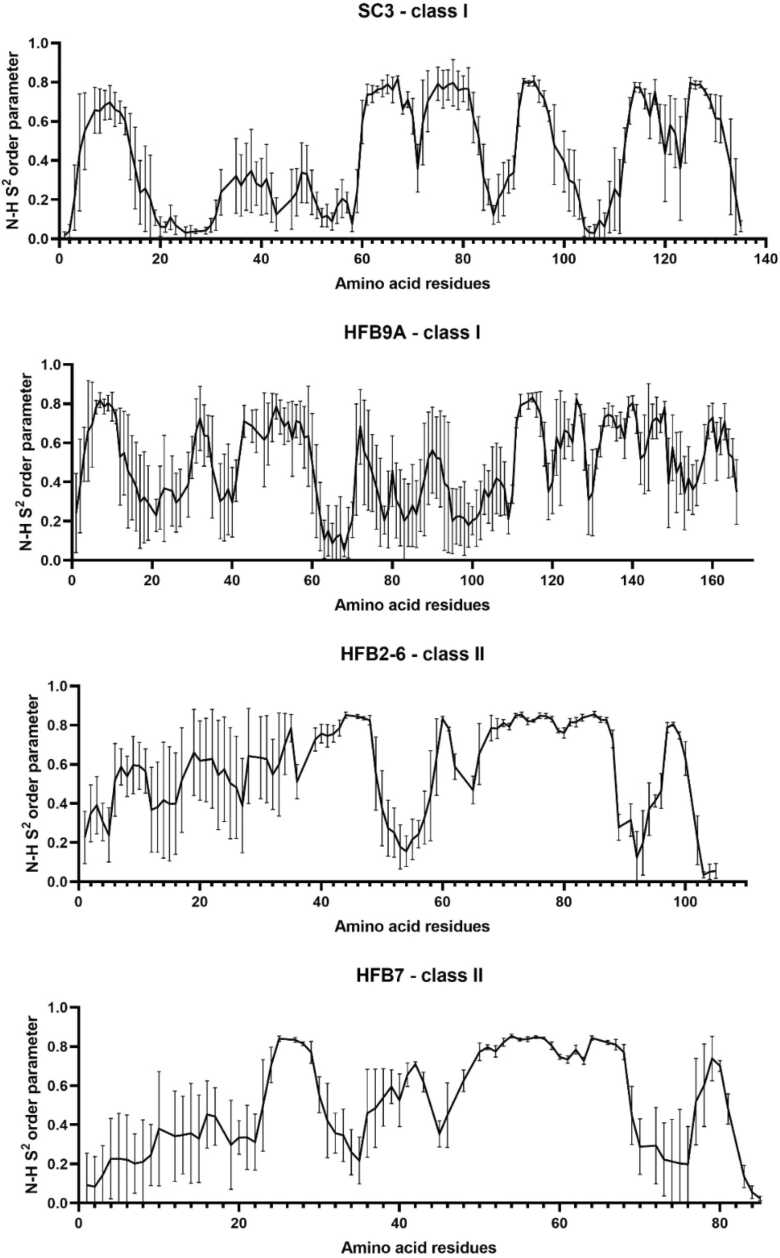
Lipari-Szabo model free order parameter (S^2^) calculation based on ‘ired’ from cpptraj. The S^2^ parameters are calculated for N-H bonds in the protein backbone. The value ranging from 0 to 1, and closer to 0 shows the amino acid dominated by internal motion i.e. high flexibility. These regions of high disorder in this correspond with observed with the RMSF analysis.

Lastly, we also report the S2 order parameters calculated on shorter window of simulation length for accurate results as suggested by [19] (Fig. 12). It calculates the amplitude of protein backbone N-H group motions based on the Lipari-Szabo model free formalism [32]. It is reported that amino acids with a small side-chain show greater flexibility and the motion of a given NH group is also affected by the surrounding amino acid residues [16]. The S2 value ranges from 0 to 1, 1 meaning complete restriction of internal motion and 0 meaning that the amino acid is completely dominated by internal motion and is very flexible. This analysis can give us yet another peek into the disordered nature of HFBs. The regions from 20 to 50 and 100–110 show values close to 0.0 and therefore, high fluctuation of the N-H bond. These two regions correspond with the 2 disordered loops discussed previously in Fig. 2. HFB9A shows a greater degree of fluctuation the N-terminus half of the sequence as also observed in the RMSF analysis. The range of error bars further support the dynamic nature of HFB9A as it evolved through the simulation. Class II HFBs, show the presence of two shorter disordered loops. For HFB2–6, amino acid residues from 50 to 60 and 90–96 show higher atomic fluctuation (Fig. 2) and lower S^2^ order values confirming the presence of highly flexible regions. The S^2^ graph for HFB7 is very similar to HFB2–6, with indications of presence of less flexible disorder loop regions. The range of first disordered loop (starting from the N-terminus) differs slightly with corresponding RMSF values. While here, the residues from 24 to 28 show highest disorder, in RMSF plot residues from 32 to 36 show a bump in atomic fluctuation values. However, the range for the second disordered loop is the same in both analyses i.e. from 70 to 80 amino acid residues.

## 4 Conclusion

The study of fungal HFBs has recently gained wider interest of the research community due to the plausible application in biotechnological and medical industry. We selected four HFB proteins belonging to the two previously categorized classes, with SC3 (*S. commune*) and HFB9A (*T. harzianum*) representing Class I, while HFB2–6 from *T*. *asperellum* ACCC30536 and HFB7 represented Class II. The experimental structures of these proteins have not been solved and not available. We firstly tried to obtain their structures using the latest AI-based AlphaFold2, which resulted in unsatisfactory folding of terminals and lower confidence scores. Therefore, these conformations were used as initial structures to run folding simulations using the advanced aMD simulation technique. Apart from representative structures, MD simulations also provide us with knowledge about their folding dynamics. Class I HFBs showed a higher degree of fluctuation and ‘disorderedness’ with the presence of 2 distinct disordered loops in SC3. HFB9A seemed to be mostly disordered with higher atomic fluctuations, close to zero S^2^ order values, loss of native contacts etc. We obtained a more compactly folded conformation in the HFB9A ensemble than what was achieved by AF2, however, the it had the least compactness in comparison to other three HFBs observed during simulations. Its representative structures show large amounts of loop regions and a partially formed β-barrel similar to NC2 from *N. crassa*. The regions of high hydrophobicity corresponded with high structural stability while highly disordered regions were mostly more hydrophilic in nature. Class II HFBs, on the other hand, were mostly stable, with definitive energy minimum states and low atomic fluctuation values and also showed a similar secondary structure profile and hydropathy patterns. Therefore, it is possible that these two classes may need to be further sub-categorized based on differences of structure, folding dynamics and hydropathy patterns. Based on aMD simulations, we also report the structural ensembles of selected HFBs solvated in the aqueous solvent. The elucidation of structures of HFBs is a stepping stone to further theoretical calculations concerning their aggregation properties and assembly at hydrophobic-hydrophilic surfaces.

## CRediT authorship contribution statement

**Marik Tamás:** Writing – review & editing, Supervision, Software. **Gufu Bonaya:** Visualization, Validation. **Agwora Derrick:** Writing – review & editing, Writing – original draft, Visualization, Validation, Software, Formal analysis. **Tyagi Chetna:** Writing – review & editing, Writing – original draft, Validation, Supervision, Software, Methodology, Formal analysis, Conceptualization. **Kredics László:** Writing – review & editing, Supervision, Software, Funding acquisition, Conceptualization. **Vágvölgyi Csaba:** Project administration, Funding acquisition. **Papp Tamás:** Project administration, Funding acquisition.

## Declaration of Competing Interest

The authors declare no conflict of interest.

We, the authors collectively and individually declare to our best ability, that we have ensured that the submission made herein does not contain any plagiarized material, content or ideas, and that all necessary attributions have been appropriately made and all copyright permissions obtained, cited and acknowledged. The manuscript has been approved by all the authors involved and is not under consideration for publication elsewhere nor has it been published elsewhere in any form and medium.
