# Supplementary material for: Structural elucidation of four fungal hydrophobins belonging to classes I and II: Results from Alphafold and accelerated molecular dynamics simulations
Source: Comput Struct Biotechnol J. 2025 Mar 12;27:1067–80. doi: 10.1016/j.csbj.2025.03.015 (PMC11957597; doi:10.1016/j.csbj.2025.03.015)
Supplement: Supplementary file 1 — Supplementary material [file mmc1.docx]

**Supplementary information**

**Structural elucidation of four fungal hydrophobins belonging to classes I and II: results from Alphafold and accelerated molecular dynamics simulations**

**Derrick Agwora^1†^, Bonaya Gufu^1^, Tamás Marik^1^, Tamás Papp^1,2^, Csaba Vágvölgyi^1,2^, László Kredics^1^, Chetna Tyagi^1^**

**^1^Department of Biotechnology and Microbiology, Faculty of Science and Informatics, University of Szeged, Szeged, Hungary**

**^2^HUN-REN-SZTE Fungal Pathomechanisms Research Group, University of Szeged, Szeged, Hungary**

**†Present address: Department of Cell Biology, Charles University, Faculty of Science, Prague, Czech Republic**

***Corresponding author:**

[**cheta231@gmail.com**](mailto:cheta231@gmail.com)**,** [**chetna.tyagi@bio.u-szeged.hu**](mailto:chetna.tyagi@bio.u-szeged.hu)


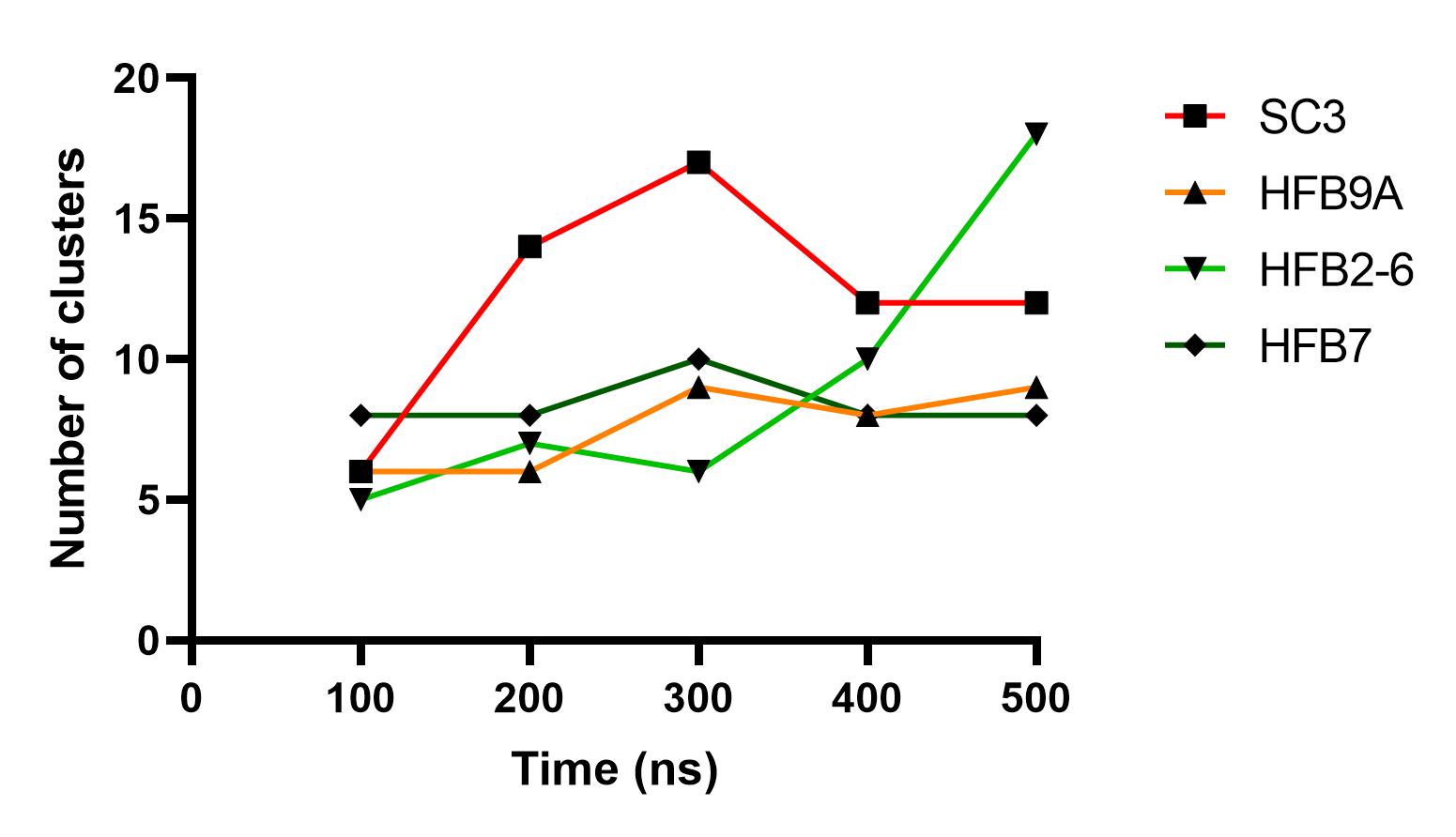


**Figure S1: Number of structural clusters obtained calculated using the peak-picking method applied to principal components (PCs) dihedral PCA for five blocks of 100 ns each. This is a self-consistency check (SCC) criterion to observe convergence of ensemble properties. The graph for #clusters plateaus after 400 ns for SC3, HFB9A and HFB7 showing that no new structurally distinct state was observed when the simulation is prolonged. HFB2-6, however, does not show convergence based on this criterion.**


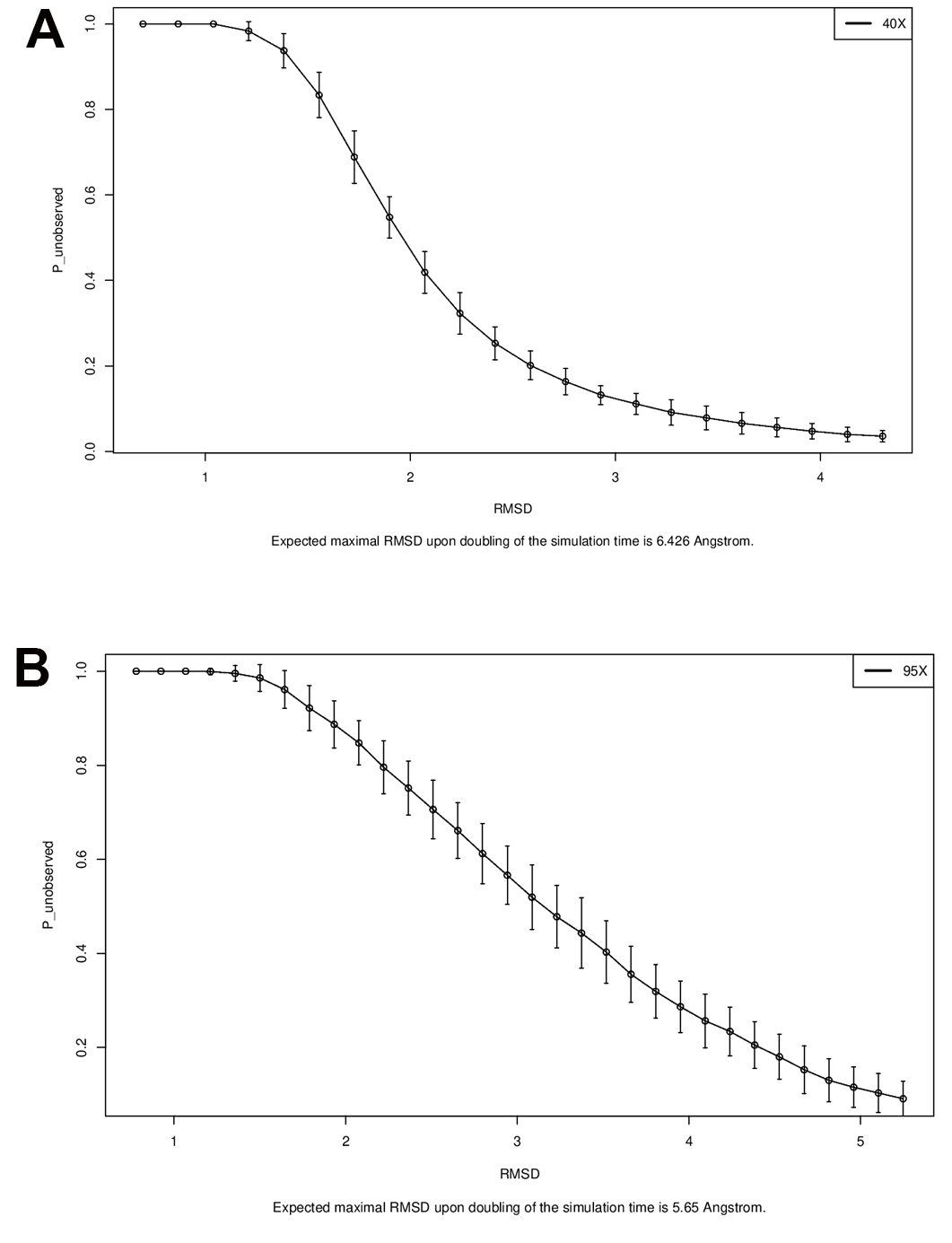


**Figure S2: Estimation of extent of sampling of (a) HFB2-6 and (b) HFB7 from aMD simulations based on Good-Turing formalism available as a tool in ‘grcarma’. The probability of unobserved species reaches 0 at the maximum RMSD (root-mean-square-deviation) value that could be observed if the simulation is extended.**
